# Regulatory mechanism of heat-active retrotransposons by the SET domain protein SUVH2

**DOI:** 10.3389/fpls.2024.1355626

**Published:** 2024-02-08

**Authors:** Xiaoying Niu, Zhiyu Ge, Hidetaka Ito

**Affiliations:** ^1^ Graduate School of Life Science, Hokkaido University, Sapporo, Hokkaido, Japan; ^2^ Faculty of Science, Hokkaido University, Sapporo, Hokkaido, Japan

**Keywords:** epigenetics, transposon, environmental stress, *Arabidopsis thaliana*, *ONSEN*

## Abstract

New transposon insertions are deleterious to genome stability. The RNA-directed DNA methylation (RdDM) pathway evolved to regulate transposon activity via DNA methylation. However, current studies have not yet clearly described the transposition regulation. *ONSEN* is a heat-activated retrotransposon that is activated at 37°C. The plant-specific SUPPRESSOR OF VARIEGATION 3–9 HOMOLOG (SUVH) family proteins function downstream of the RdDM pathway. The SUVH protein families are linked to TE silencing by two pathways, one through DNA methylation and the other through chromatin remodeling. In this study, we analyzed the regulation of *ONSEN* activity by SUVH2. We observed that *ONSEN* transcripts were increased; however, there was no transpositional activity in Arabidopsis *suvh2* mutant. The *suvh2* mutant produced siRNAs from the *ONSEN* locus under heat stress, suggesting that siRNAs are involved in suppressing transposition. These results provide new insights into the regulatory mechanisms of retrotransposons that involve siRNA in the RdDM pathway.

## 1 Introduction

Plants and animals must cope with various environmental stressors. Environmental stress can activate transposable elements (TEs), and new insertions are inherited by the next generation of germ cells (Grandbastien et al., 1997; Oliver and Greene, 2009) contributing to genome evolution in plants (Oliver and Greene, 2009; Bennetzen and Wang, 2014). TEs are categorized into two main classes based on their mode of transposition: class I and II transposons (Bourque et al., 2018). Class I transposons are known as retrotransposons, and their transposition activity requires an RNA intermediate. Long terminal repeat (LTR) and non-LTR retrotransposons (long interspersed nuclear elements [LINEs] and short interspersed nuclear elements [SINEs]) are the two main classes of retrotransposons (Wicker et al., 2007). The transposition activity of the LTR retrotransposon begins at the promoter within the 5’ LTR, where the TE is first transcribed into RNA by RNA polymerase II. In the cytoplasm, the RNA is reverse-transcribed into cDNA and reintroduced into the nucleus, and, finally, the cDNA is reincorporated into the host DNA (Havecker et al., 2004). In contrast, most class II elements accomplish transposition activity by excising themselves and thus jumping to a new gene location (Wells and Feschotte, 2020). TEs regulate gene activity by transpositional insertions into or close to genes, manifesting either through the direct disruption of gene transcription or by indirectly affecting gene expression through epigenetic modifications (Niu et al., 2022a; Berthelier et al., 2023).

TEs are regulated through epigenetic modifications, such as two representative epigenetic processes: DNA methylation and histone modification (Lippman et al., 2004; Slotkin and Martienssen, 2007; Mhiri et al., 2022). In plants, *de novo* DNA methylation is induced by RNA-directed DNA methylation (RdDM), mediated by small interfering RNAs (siRNAs) and scaffolding RNAs (Gallego-Bartolome, 2020). RdDM is classified into canonical and non-canonical pathways (Matzke and Mosher, 2014). In the canonical RdDM pathway, Pol IV (RNA polymerase IV) and Pol V (RNA polymerase Pol V) are essential for the RdDM process and serve the production of 24 nt siRNA and long non-coding RNA (lncRNAs) (Onodera et al., 2005; Wierzbicki et al., 2008). Previous reports have shown that there are ten different suppressors of variegation 3–9 homolog (SUVH) family proteins in *Arabidopsis thaliana* (Naumann et al., 2005), of which SUVH2 and SUVH9 are closely associated with DNA methylation activities in RdDM (Johnson et al., 2008). SUVH2 and SUVH9 bind to methylated DNA and promote the recruitment of Pol V to the target locus to produce long non-coding RNAs by interacting with the DDR (DRD1-DMS3-RDM1; defective in RNA-directed DNA methylation 1 [drd1], defective in meristem silencing 3 [DMS3], and RNA-directed DNA methylation 1 [RDM1]) and MORC (microrchidia) complex (Kanno et al., 2004; Johnson et al., 2008; Ream et al., 2009; Law et al., 2010; Zhong et al., 2012; Brabbs et al., 2013; Liu et al., 2014).

*ONSEN* is an LTR retrotransposon defined in *A. thaliana* and is usually activated by heat stress (Ito et al., 2011). Previous studies have shown that some RdDM pathway mutants subjected to heat stress show transcriptional activation of *ONSEN* (Hayashi et al., 2020; Niu et al., 2022b). siRNAs are involved in transcriptional repression processes, especially in TEs (Moses et al., 2010). In the *nrpd1* mutant (siRNA biogenesis deficient mutant), *ONSEN* transcripts and extrachromosomal DNA levels increased, and transgenerational transposition was observed in the next generation (Ito et al., 2011). However, our understanding of *ONSEN* transposition repression has yet to be profound. This study focused on the contributions of SUVH2 and SUVH9 to the regulatory mechanism of *ONSEN*.

## 2 Materials and methods

### 2.1 Plant material

*Arabidopsis thaliana* accession Columbia (Col-0) was used as a wild-type control. The *suvh2* (*SALK_079574*) and *suvh9* (*SALK_048033*) mutants were obtained from the ABRC stock center, and the *nrpd1* (*C366150*) mutant was obtained from Ohio State University. *Suvh2* mutants were crossed with *suvh9* or *nrpd1* mutants to generate *suvh2/9* and *suvh2/nrpd1* double mutants, respectively. All mutants were of the Col-0 background.

### 2.2 Growth conditions

Seeds sterilized in a 1:1 solution of sodium hypochlorite and 0.04% Triton X-100 were planted in 0.5× Murashige and Skoog (MS) medium. Seeds were sowed at 4°C (dark conditions) for 2 days and then cultured in 0.5x MS medium or potting (soil: vermiculite = 1:7) at 21°C under continuous light.

### 2.3 Heat-stress treatment

Seven-day-old seedlings grown in 0.5× MS medium at 21°C under continuous light were subjected to heat stress at 37°C. For Southern blot experiments, we allowed them to recover to 21°C for two days before transferring them to the soil because the 7-day-old plants we analyzed would be weakened if transplanted directly into the soil immediately after heat treatment. Then, the seedlings were transferred to the soil at 21°C under continuous light. Progeny seeds were directly planted in the soil at 21°C under continuous light.

### 2.4 Southern blotting

DNA was extracted from 3-week-old plants using a Nucleon PhytoPure DNA Extraction Kit (Cytiva, Tokyo, Japan). The 2.4 µg of extracted DNA was processed with the restriction enzyme *EcoR*V overnight at 37°C, and the enzyme-treated DNA was purified by ethanol precipitation. DNA was electrophoresed on a 1% agarose gel for 24 h at 20 V. The DNA was transferred onto a Biodyne B Nylon Membrane (Thermo Fisher Scientific, Waltham, Massachusetts, USA) overnight. PCR product for the probe was generated by TaKaRa Ex Taq (TaKaRa, Kusatsu, Japan). The 577 bp of the predicted ORF of *ONSEN* was used as a probe. The PCR primers are shown in **Supplementary Table 1**. Hybridization signals were detected in a high SDS hybridization buffer using the Megaprime DNA Labeling System (Cytiva, Tokyo, Japan) with radioisotope-labeled probes.

### 2.5 Quantitative analysis

For qRT-PCR, total RNA was extracted using the TRI reagent (Sigma-Aldrich, St. Louis, Missouri, USA). After treatment with RQ1 Rnase-Free Dnase (Promega, Madison, Wisconsin, USA), RNA was reverse-transcribed using the ReverTra Ace qPCR RT Kit (TOYOBO, Osaka, Japan) to synthesize cDNA. The total DNA was extracted using the Nucleon PhytoPure DNA Extraction Kit (Cytiva, Tokyo, Japan) for qPCR. The Ct method was used to determine expression, and the expression of the 18S rRNA gene was used as an internal control (Schmittgen and Livak, 2008). qPCR amplification was performed with the appropriate primers (**Supplementary Table 1**).

### 2.6 DNA methylation analysis

The DNA used for bisulfite sequencing was obtained from 7-day-old seedlings. Total DNA was bisulfite-converted and desulfated using a MethylCode Bisulfite Conversion Kit (Invitrogen, Waltham, Massachusetts, USA). The target fragments were amplified by PCR (40 cycles, 98°C for 10 s, 55°C for 30 s, 72°C for 1.5 min), and the PCR products were cloned. PCR amplification was performed with the appropriate primers (**Supplementary Table 1**). Twenty-four clones were sequenced, and the results were analyzed using the MEGA-X software and the Cymate website to detect DNA methylation levels.

### 2.7 Formaldehyde-assisted isolation of regulatory elements

Seven-day-old seedlings (0.3 g) were cross-linked in a 1% formaldehyde solution (Thermo Fisher Scientific, Waltham, Massachusetts, USA) under vacuum for 10 min (2 min + 8 min). The cross-linking was quenched by adding glycine at a final concentration of 0.125 M for 5 min under vacuum. The samples were ground to powder with liquid nitrogen, and separation buffer 1 (0.4 M sucrose, 10 mM Tris-HCl, pH 8, 10 mM MgCl2, 5 mM β-mercaptoethanol, and protease inhibitor [Roche, Basel, Switzerland]) was added, followed by filtration using Miracloth (Millipore, Burlington, Massachusetts, USA) to remove cell debris. After centrifugation at 2000 g for 20 min at 4°C, precipitated nuclei were dispersed in isolation buffer 2 (0.25 M sucrose, 10 mM Tris-HCl, pH 8, 10 mM MgCl2, 1% Triton X-100, 5 mM β-mercaptoethanol, and protease inhibitor [Roche, Basel, Switzerland]). After centrifugation at 13,000 rpm for 20 min at 4°C, the precipitated nuclei were re-dispersed in Isolation Buffer 2. Add Isolation Buffer 3 (0.25 M sucrose, 10 mM Tris-HCl, pH 8, 10 mM MgCl2, 1% Triton X-100, 5 mM β-mercaptoethanol, and protease inhibitor [Roche, Basel, Switzerland]) to keep the solution stratified and centrifuge for 60 min at 4°C at 13000 rpm. The precipitates were resuspended using 0.3 mL of SDS nuclear lysis buffer (50 mM Tris-HCl pH 8, 10 mM EDTA, 1% SDS, and protease inhibitor cocktail). The chromatin was sheared using Covaris M220 (Covaris, Woburn, Massachusetts, USA). Suspensions were centrifuged at 13,000 rpm for 10 min at 4°C to obtain a supernatant. DNA was purified using the phenol-chloroform method. The abundance of DNA fragments relative to the input DNA was determined using qPCR. qPCR amplification was performed with the appropriate primers (**Supplementary Table 1**). For input DNA treatment, 2 µL of supernatant was mixed with 198 µL of extraction buffer (10 mM Tris-HCl pH 8, 0.3 M NaCl, 5 mM EDTA, 0.5% SDS) and incubated overnight at 65°C to reverse formaldehyde cross-linking. The de-crosslinked DNA was purified using the phenol-chloroform method after treatment with Rnase and proteinase K. Input DNA was used as an internal control to identify the non-crosslinked portion of the condensed genomic region (NDR).

### 2.8 Cytology

The leaves of 7-day-old plants were fixed overnight with ethanol: acetic acid (3:1). Leaves were treated with the enzymes (boehmotoxin [Yakult Pharmaceutical Industries, Yakult Pharmaceutical Industries], pectinase [Kyowa Chemical Products, Tokyo, Japan], and cytochromes [Sigma-Aldrich, St. Louis, Missouri, USA] [1% (v/v) in citrate buffer]) at 37°C for 2 h. The leaves were placed on a slide on a heating plate at 45°C for 30 s, and the leaves were simultaneously torn with a needle to disperse the tissue in 45% acetic acid. After the acetic acid had evaporated, a fixative was added, and the slides were dried. Staining was performed by adding 4′,6-diamidino-2-phenylindole (DAPI) (Vector Laboratories, Newark, California, USA).

### 2.9 Northern blotting

Total RNA was extracted using the TRI reagent (Sigma-Aldrich, St. Louis, Missouri, USA). Low-molecular-weight RNA was purified by ethanol precipitation and dissolved in 100% formamide. RNA samples (1400ng) were denatured at 65°C for 5 min, and electrophoresis was performed on a 15% PAGE gel in 0.5× TBE buffer, run at 50 V for 20 min to make the dyes move at the same rate, and run at 200 V and 500 mA for 3 h. The RNA was transferred onto a Hybond-N+ hybridization membrane (Sigma-Aldrich, St. Louis, Missouri, USA) overnight and hybridized with a DIG-labeled RNA probe at 40°C overnight. We used synthetic oligonucleotides containing the T7 RNA polymerase promoter and the MRGA shortscript kit for generating probe (Thermo Fisher Scientific, Waltham, Massachusetts, USA). The PCR primers for the probe are shown in **Supplementary Table 1**. DIG-labeled RNA probes were synthesized using the DIG RNA Labeling Mix (Roche, Basel, Switzerland). Hybridized signals were detected using anti-dig (Roche, Basel, Switzerland) and CDP Star (Roche, Basel, Switzerland) using a LAS3000.

## 3 Results

### 3.1 Deletion of SUVH2 results in massive activation of *ONSEN* transcription

**Figure 1:**
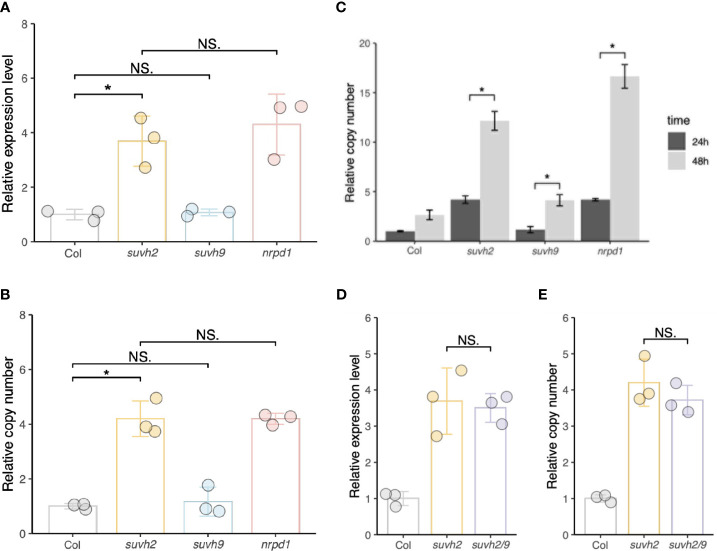
SUVH2 is essential for the transcriptional repression of *ONSEN* but not SUVH9. **(A)** and **(B)** Relative expression levels **(A)** and relative copy number **(B)** of *ONSEN* in wild-type, *suvh2*, *suvh9*, and *nrpd1* mutants at 24 h heat stress. **(C)** Relative copy number of *ONSEN* in wild type, *suvh2*, *suvh9*, and *nrpd1* at 24 h and 48 h heat stress. **(D)** and **(E)** Relative expression levels **(D)** and relative copy numbers **(E)** of *ONSEN* in wild-type, *suvh2*, and *suvh2/9* mutants at 24 h heat stress. Asterisks indicate significant differences between the two groups (Student’s t-test, P ≤0.05). NS indicates no significant differences between the two groups (Student's t-test, P >0.05).

We investigated transcript levels and copy numbers (extrachromosomal cDNA levels) of *ONSEN* in WT, *suvh2*, *suvh9*, and *nrpd1*. We observed that after 24 h of heat stress, the transcript level and copy number of *ONSEN* in *suvh2* were significantly increased compared to those in the WT and exhibited approximately the same level as in *nrpd1* (**Figures 1A, B**), suggesting that deletion of *suvh2* released the transcriptional silencing of *ONSEN*. Consistent with previous findings, 48 h of heat treatment resulted in a further increase in *ONSEN* copy compared with 24 h of heat treatment (**Figure 1C**). In *suvh9*, *ONSEN* exhibited the same transcript levels and copy number as in the WT (**Figures 1A, B**). To determine the synergistic effect of SUVH2 and SUVH9 on the RdDM pathway, we investigated the transcript and copy number levels of *ONSEN* in the *suvh2/9* double mutant. However, *ONSEN* did not exhibit higher transcript levels or copy numbers in *suvh2/9* than in *suvh2* (**Figures 1D, E**), suggesting that SUVH9 was not involved in the transcriptional repression of *ONSEN* by RdDM.

### 3.2 The transposition of *ONSEN* is infrequent in *suvh2* mutants

**Figure 2:**
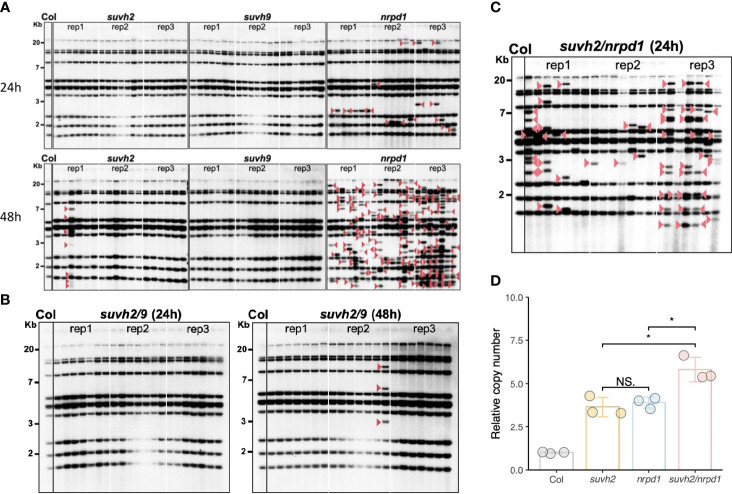
In *suvh2* mutants, the frequency of *ONSEN* transposition is low. **(A)** Southern blot results show transgenerational transposition of *ONSEN* in *suvh2*, *suvh9*, and *nrpd1* under heat stress of 24 h (top) or 48 h (bottom). **(B)** New insertions of *ONSEN* in *suvh2/9* under heat stress conditions at 24 h (left) or 48 h (right). The leftmost lane of each graph is the WT under non-stress conditions. For each mutant, three plants were subjected to heat stress (24 h or 48 h), and seven progenies were considered for *ONSEN* transposition analysis. Red arrow head indicates *ONSEN* insertions. **(C)** The next generation of s*uvh2/nrpd1* mutants after 24 h heat stress was analyzed by Southern blot to examine the transgenerational transposition of *ONSEN*. The leftmost lane of each graph is the wild type under non-stress conditions. Three homozygous mutant plants were heat stressed, and seven progenies were considered for *ONSEN* transposition analysis for each individual. Red triangles indicate insertions of *ONSEN*. **(D)** Relative copy number of *ONSEN* in wild-type, *suvh2*, *nrpd1*, and *suvh2/nrpd1* under 24 h of heat stress. Asterisks indicate significant differences between the two groups (Student’s t-test, P ≤0.05). NS indicates no significant differences between the two groups (Student’s t-test, P >0.05).

To investigate whether the disruption of SUVH2 and SUVH9 affects *ONSEN* transposition, we investigated the transgenerational transposition of *ONSEN* in *suvh2* and *suvh9* mutants. Previous studies have shown that transposition frequency correlates with the duration of heat stress; *suvh2* and *suvh9* were subjected to heat stress for 24 and 48 h, respectively. Southern blot analysis showed that *ONSEN* transposition was observed in the *suvh2* mutant after 48 h of heat stress. In contrast, no transgenerational transposition occurred after 48 h of heat stress in the *suvh9* mutant (**Figure 2A**). This suggested that SUVH2 regulates *ONSEN* transposition. However, the frequency of *ONSEN* transposition in *suvh2* was much lower than *nrpd1* (**Figure 2A**). Because SUVH2 and SUVH9 are partially non-redundant in RdDM (Kuhlmann and Mette, 2012), we investigated *ONSEN* transposition in *suvh2/9*. The results showed that no new *ONSEN* insertion was observed in *suvh2/9* after 24 h of heat treatment; however, a new insertion was observed after 48 h of heat stress (**Figure 2B**). The frequency of *ONSEN* transposition was low in *suvh2/9* cells (**Figure 2B**), suggesting that SUVH2 and SUVH9 do not synergistically suppress *ONSEN* transposition. No *ONSEN* transposition was observed under heat stress in *suvh9* (**Figure 2A**), suggesting that SUVH9 was not directly involved in suppressing *ONSEN* transposition. Although *ONSEN* cDNA accumulated in *suvh2* after heat stress, it could not be inserted into new genomic loci. Next, we investigated the activity of *ONSEN* in *suvh2/nrpd1* double mutants. In the *suvh2/nrpd1* double mutant after 24 h of heat treatment, *ONSEN* exhibited a higher transposition frequency than *suvh2* (**Figure 2C**). However, the transposition frequency is similar to that in *nrpd1* (**Figure 2C**). Also, we analyzed the transposition and cDNA levels of *ONSEN* in *suvh2/nrpd1* under 48h HS. The results showed that *ONSEN* in *suvh2/nrpd1* after 48h heat treatment showed higher transposition frequency and cDNA levels. The copy number of *ONSEN* was significantly higher in *suvh2/nrpd1* than in *suvh2* or *nrpd1* (**Figure 2D**). This suggests that SUVH2 and NRPD1 synergistically affect the regulation of *ONSEN* transcription, but not transposition.

### 3.3 DNA hypomethylation was independent of the transposition of *ONSEN* in *suvh2*

**Figure 3:**
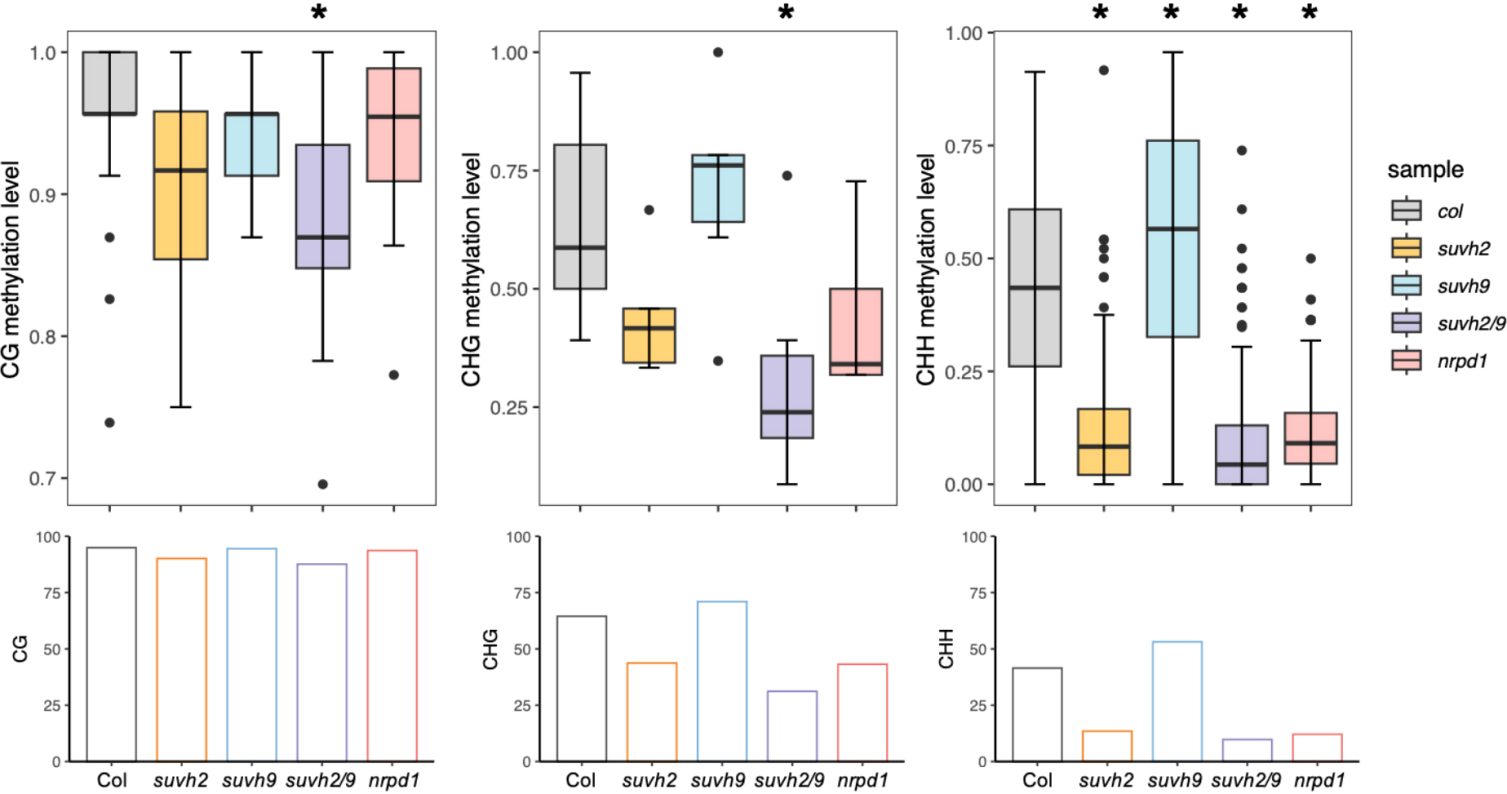
SUVH2 is essential for the establishment of DNA methylation on *ONSEN*. Box plots (top) showing CG (left), CHG (middle), and CHH (right, H = A, T, C) methylation levels of *ONSEN* (*At1g11265*) in wild-type, *suvh2*, *suvh9*, *suvh2/9*, and *nrpd1* mutants under non-stress conditions. Bar graphs (bottom) show the average levels of CG (left), CHG (middle), and CHH (right, H = A, T, C) methylation of *ONSEN* in each mutant. Asterisks indicate significant differences compared to Col (Student’s t-test, P > 0.05).

SUVH2 and SUVH9 are essential for DNA methylation (Liu et al., 2014). To determine what suppresses *ONSEN* transposition in *suvh2*, we investigated DNA methylation levels of the *ONSEN* in the SUVH mutant. We observed that the level of CHH methylation in *ONSEN* was significantly reduced in *suvh2* compared to WT (**Figure 3**; **Table 1**), suggesting that SUVH2 is essential for establishing CHH DNA methylation in *ONSEN*. This result was similar to that obtained for the single-copy SINE *AtSN1* (Johnson et al., 2008). The loss of SUVH2 and SUVH9 resulted in lower CHH methylation levels (**Figure 3**; **Table 1**), suggesting that SUVH2 and SUVH9 have synergistic effects in regulating methylation. However, the level of CHH methylation in *ONSEN* was significantly higher in *suvh9* mutants than in the WT (**Figure 3**; **Table 1**). The *ONSEN* promoter is present in the LTR region, and DNA methylation is present only in the CHH contexts. The CHH hypermethylation of *ONSEN* in *suvh9* may be responsible for the inability of *ONSEN* to be transcribed at a high level. Heat stress did not change the DNA methylation levels of the *ONSEN* region in *suvh2* (**Supplementary Figure 1**), which is consistent with our previous findings (Niu et al., 2022b). In addition, the DNA methylation pattern of *ONSEN* in *suvh2* was nearly identical to that of *nrpd1* (**Figure 3**), indicating that DNA hypomethylation released transcriptional silencing of *ONSEN* but did not affect transposition.

**Table 1:** Results of significant difference analysis of methylation data and asterisks indicate significant differences in **Figure 3** (Student’s t-test, P < 0.05).

	group1	group2	p	p.adj	p.signif
CG	col	*suvh2*	0.0928273	0.62	ns
	col	*suvh9*	0.22078234	0.88	ns
	col	*suvh2/9*	0.00222573	0.022	**
	col	*nrpd1*	0.09985313	0.62	ns
	*suvh2*	*suvh9*	0.39217267	0.9	ns
	*suvh2*	*suvh2/9*	0.0886009	0.62	ns
	*suvh2*	*nrpd1*	0.39921964	0.9	ns
	*suvh9*	*suvh2/9*	0.00259797	0.023	**
	*suvh9*	*nrpd1*	0.30059472	0.9	ns
	*suvh2/9*	*nrpd1*	0.06608209	0.53	ns
	group1	group2	p	p.adj	p.signif
CG	col	*suvh2*	0.0928273	0.62	ns
	col	*suvh9*	0.22078234	0.88	ns
	col	*suvh2/9*	0.00222573	0.022	**
	col	*nrpd1*	0.09985313	0.62	ns
	*suvh2*	*suvh9*	0.39217267	0.9	ns
	*suvh2*	*suvh2/9*	0.0886009	0.62	ns
	*suvh2*	*nrpd1*	0.39921964	0.9	ns
	*suvh9*	*suvh2/9*	0.00259797	0.023	**
	*suvh9*	*nrpd1*	0.30059472	0.9	ns
	*suvh2/9*	*nrpd1*	0.06608209	0.53	ns
	group1	group2	p	p.adj	p.signif
CHH	col	*suvh2*	1.29E-20	7.70E-20	****
	col	*suvh9*	0.00037618	0.0011	***
	col	*suvh2/9*	4.64E-24	3.20E-23	****
	col	*nrpd1*	3.37E-19	1.70E-18	****
	*suvh2*	*suvh9*	9.21E-29	8.30E-28	****
	*suvh2*	*suvh2/9*	0.02640555	0.053	*
	*suvh2*	*nrpd1*	0.46133787	0.46	ns
	*suvh9*	*suvh2/9*	1.37E-31	1.40E-30	****
	*suvh9*	*nrpd1*	6.29E-28	5.00E-27	****
	*suvh2/9*	*nrpd1*	6.29E-05	0.00025	****

Student’s t-test, ns means P>0.05, * means P ≤0.05, ** means P ≤0.01, *** means P <0.001, **** means P≤0.0001.

### 3.4 Chromatin condensation was independent of the activation of *ONSEN*

**Figure 4:**
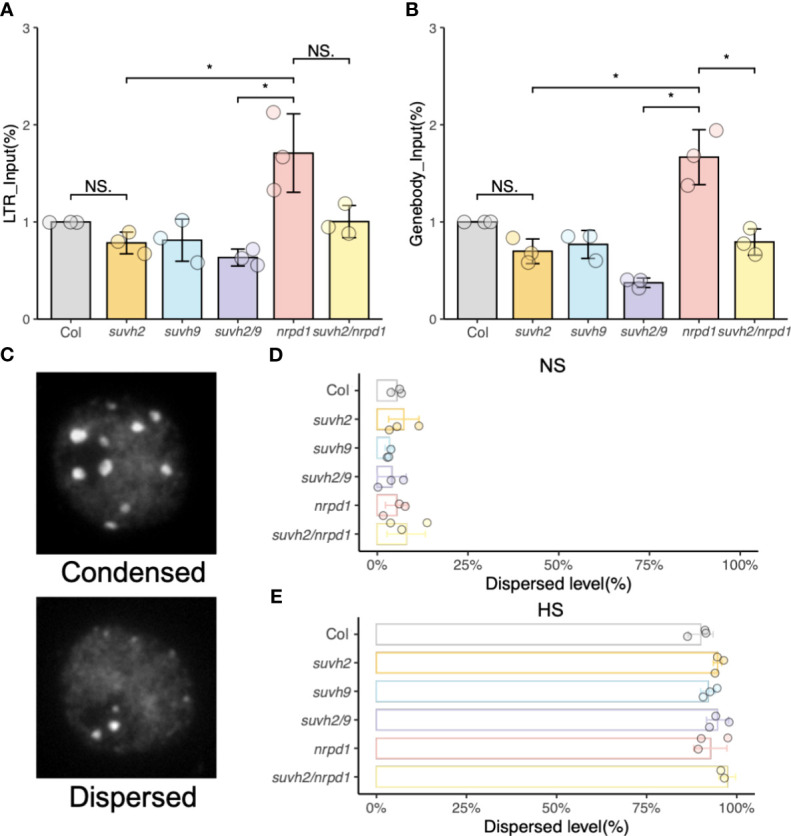
Analysis of open chromatin. **(A)** and **(B)** FAIRE-qPCR results show the level of open chromatin in a small region of LTR **(A)** and genebody **(B)** on *ONSEN*. **(C)** Representative images of nuclei under DAPI staining. **(D)** and **(E)** Percentage of chromatin in two (condensed or dispersed) states in wild-type, *suvh2*, *suvh9*, *suvh2/9*, *nrpd1*, and *suvh2/nrpd1* under non-stress (NS) **(C)** and 48 h heat stress (HS) **(D)** (n = 150). Asterisks indicate a significant difference between the two groups (Student’s t-test, P <0.05). NS indicates no significant difference between the two groups (Student’s t-test, P ≥0.05).

Chromatin repression causes transgene silencing in a non-DNA methylation-dependent manner. (Mittelsten Scheid et al., 2002). FAIRE-qPCR was used to examine the status of chromatin condensation in the LTR, or gene body region, of *ONSEN*. The levels of open chromatin in the LTR and gene body of *ONSEN* in *suvh2* and *suvh2/9* were much lower than those in *nrpd1* (**Figures 4A, B**). Since the promoter of *ONSEN* exists within LTRs, we concluded that the open chromatin of *ONSEN* does not directly correlate with the transcriptional repression of *ONSEN*. The open chromatin of the *ONSEN* gene body region appeared to be lower in *suvh2/nrpd1* double mutants than in *nrpd1* (**Figure 4B**). As heat stress activates *ONSEN*, producing extrachromosomal cDNAs, FAIRE of the *ONSEN* region after heat treatment is challenging. DAPI staining was used to investigate the condensation of heterochromatin in the nuclei of the mutants. We categorized heterochromatin as condensed or dispersed (**Figure 4C**). Heat stress loosened the heterochromatin; dispersed nuclei showed similar proportions across mutants with or without heat stress treatment (**Figures 4D, E**). These results imply that the chromatin state may not affect *ONSEN* transcription or transposition.

### 3.5 The siRNA regulates *ONSEN* transcription and transposition in *suvh2*

**Figure 5:**
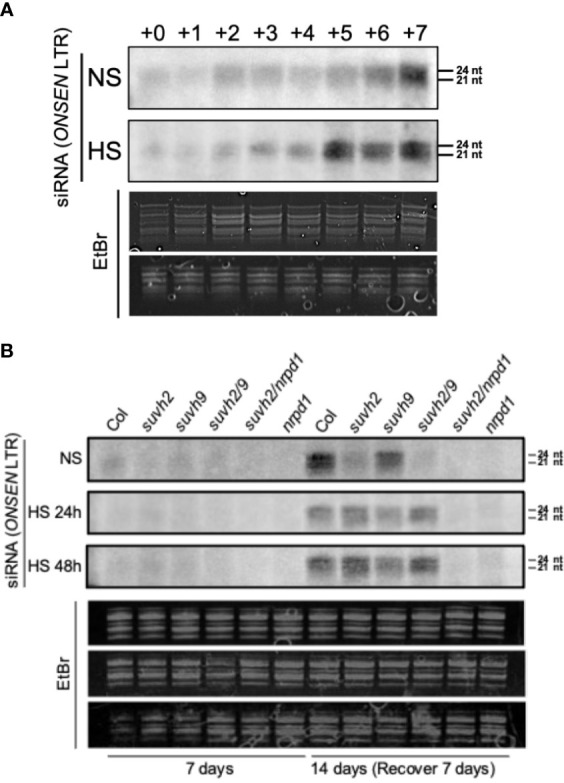
Northern blot analysis to investigate siRNA accumulation from the *ONSEN* LTR. **(A)** Levels of siRNA accumulation in WT under non-stress (NS) or heat stress (HS) conditions. The numbers above represent the accumulation levels of siRNAs in one-week-old seedlings placed under non-stress (NS) or heat stress (HS) conditions (37°C) for 24 h (+0) and recovery at normal conditions (21°C) for one day (+1), two days (+2)…seven days (+7) after heat stress treatment. **(B)** Accumulation levels of siRNAs in wild-type, *suvh2*, *suvh9*, *suvh2/9*, *nrpd1*, and *suvh2/nrpd1* in 7-week-old seedlings treated with non-stress (NS) or heat stress (HS) (24 h or 48 h) and recovered at normal conditions (21°C) for seven days (+7) after treatment.

Pol IV is a significant factor in siRNA synthesis, and siRNAs are involved in *ONSEN*’s transcriptional repression regulation. Therefore, we explored the siRNA accumulation of *ONSEN* in each mutant. First, we investigated the accumulation of siRNAs in the LTR of *ONSEN* in WT plants under non-stress and heat-stress conditions. The results showed that siRNA was not produced immediately after heat stress and gradually accumulated in recovery at 21 degrees (**Figure 5A**). siRNA was produced in the WT plants under non-stressed conditions, and the amount of siRNA gradually increased over time (**Figure 5A**).

**Figure 6:**
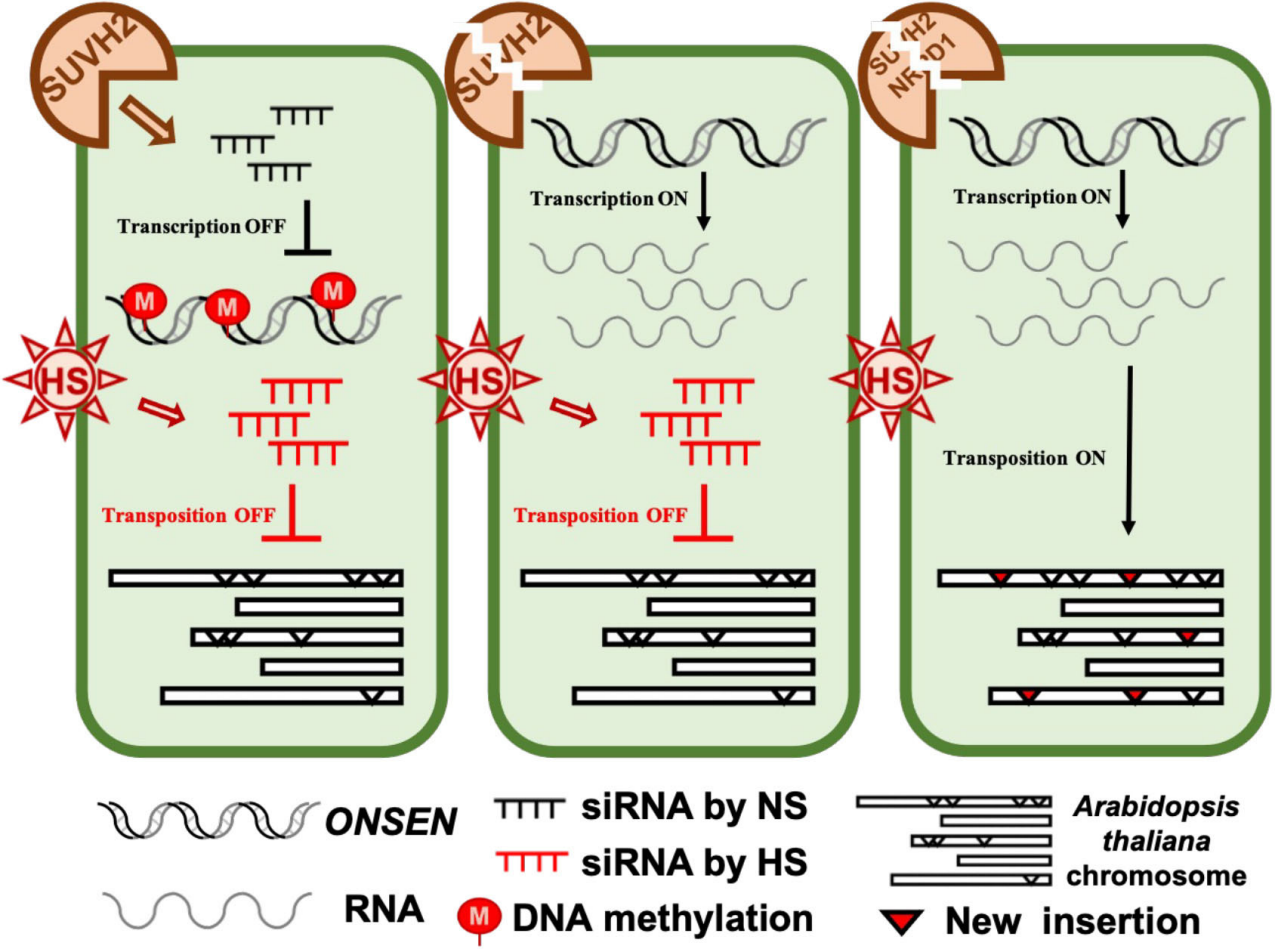
The model of SUVH2 involves *ONSEN* regulation. In the wild-type, intact RdDM can produce siRNAs to suppress *ONSEN* transcription. In the *suvh2* mutant, the RdDM pathway is disrupted, releasing *ONSEN* transcription by heat stress. Under heat stress, plants bypass the SUVH2-dependent pathway to produce new siRNAs, and these siRNAs produced by heat stress prevent *ONSEN* from transposing into other sites on the chromosome. In the *suvh2/nrpd1* double mutant, siRNA could not be synthesized under heat stress conditions releasing of transpositional silencing of *ONSEN*.

Because large amounts of siRNA were not produced immediately after heat stress, we investigated the accumulation levels of siRNA after heat stress and after 7 days of recovery. Accumulation of siRNA was observed in WT, *suvh2*, *suvh9*, and *suvh2/9* plants recovered at 21 degrees for 7 days after both 24-h and 48-h heat treatments (**Figure 5B**). As expected, we did not detect siRNA production in plants deficient in NRPD1 (**Figure 5B**). This suggests that the deletion of SUVH2 and/or SUVH9 did not completely disrupt siRNA production, inhibiting *ONSEN* transposition. In contrast, accumulation of siRNAs was observed in *suvh9* without heat stress, whereas siRNAs did not appear in *suvh2* and *suvh2/9* (**Figure 5B**). Because heat stress did not increase *ONSEN* transcription in *suvh9* (**Figure 1A**), we suggest that siRNA produced under non-stress conditions may make it difficult for *ONSEN* to be transcribed when subjected to heat stress. Since the deletion of SUVH2 reduced the production of *ONSEN* siRNA under non-stress conditions (**Figure 5B**), thereby diminishing siRNA involvement in heat stress, which could lead to the disruption of the siRNA-dependent transcriptional repression of *ONSEN*, However, *suvh2* mutant plants under heat stress showed similar amount of siRNAs to WT plants, and we propose that it is precisely the siRNAs produced after heat stress that result in the inhibition of *ONSEN* transposition in *suvh2* mutants (**Figure 6**).

## 4 Discussion

RdDM is essential for plant resistance to biotic and abiotic stresses (Erdmann and Picard, 2020). Downstream of the RdDM pathway, SUVH2 and SUVH9 are significant for the association of Pol V with chromatin (Johnson et al., 2014). In this study, we investigated the inhibition of *ONSEN* activity by SUVH2 and SUVH9. Our results show that SUVH2 represses a large amount of *ONSEN* transcription. In contrast, SUVH9 did not transcriptionally repress *ONSEN* (**Figure 2A**). *AtSN1*, a SINE retrotransposon found in *A. thaliana*, is a model target for studying RdDM and its transcriptional silencing. Liu et al. proposed that SUVH2 and SUVH9 have redundant functions in transcriptional silencing and observed that *AtSN1* expression was increased in the *suvh2/9* double mutant and not in the *suvh2* or *suvh9* single mutant (Liu et al., 2014). This result differed from the expression of *ONSEN*, and we hypothesized that SUVH2 and SUVH9 are partially non-redundant when involved in the transcriptional repression of *ONSEN*. Another possibility is that SUVH9 is not involved in the transcriptional repression of *ONSEN*.

We observed the new *ONSEN* insertions in the progeny of the 48-h heat-stressed *suvh2* (**Figure 1A**). In contrast, *ONSEN* transposition was suppressed in the plants after 24 h of heat stress (**Figure 1A**). The copy number of *ONSEN* was not reduced in *suvh2* compared to that in *nrpd1*, in which a high frequency of *ONSEN* transposition occurred (**Figures 1A**, **2C**). This may be due to the post-transcriptional regulation of *ONSEN* in *suvh2*.

SUVH2 and SUVH9 lack the SET post-structural domain, and although they do not have histone methyltransferase activity, they can participate in RdDM by binding methylated DNA through their SET and RING-associated (SAR) domains (Johnson et al., 2008). The level of DNA methylation in the LTR region of *ONSEN* was lost in SUVH2-deficient plants, especially CHH methylation (**Figure 3**). A related report on SDC showed that a lack of either *suvh2* or *suvh9* results in the loss of CHH methylation (Johnson et al., 2008). This contrasts with our finding that there was a slight increase instead of a decrease in DNA methylation in the *ONSEN* region of *suvh9* (**Figure 3**). The high DNA methylation level of *ONSEN* in *suvh9* is responsible for its low transcriptional activity. *ONSEN* showed a similar pattern of DNA methylation in *suvh2* and *nrpd1* (**Figure 3**). In conclusion, we suggest that the transcriptional activity of *ONSEN* depends on the level of DNA methylation.

Heterochromatin is enriched with transposable elements (Marsano and Dimitri, 2022). Although chromatin accessibility is associated with establishing DNA methylation (Zhong et al., 2021), it has been suggested that chromatin remodeling factors may occur independently of the DNA methylation process. For example, Morpheus Molecue 1 (MOM1) is required to silence repetitive heterochromatin sequences and is involved in epigenetic modification in a DNA methylation-independent manner (Amedeo et al., 2000). Similarly, AtMORC1 and AtMORC6 are involved in the RdDM pathway by controlling the decondensation of heterochromatin around filaments and not through DNA methylation (Moissiard et al., 2012). Jing et al. identified SUVH9 as a linker between MORC-mediated chromatin remodeling (Jing et al., 2016). We speculated that SUVH9 might be involved in silencing *ONSEN* via a non-DNA methylation pathway. Deletion of SUVH2 and/or SUVH9 did not result in open chromatin in the *ONSEN* LTR or gene body (**Figures 4A, B**). SUVH9 plays a minimal role in *ONSEN* silencing. In addition, the results of DAPI staining suggested that heat stress leads to the decondensation of heterochromatin. However, heat-induced decondensation of heterochromatin was not *nprd1* mutant-specific (**Figures 4D, E**). These results suggest that heterochromatin decondensation does not directly affect the transgenerational transposition of *ONSEN*. Open chromatin may be essential for transposition activity. However, it has been challenging to determine open chromatin in the promoter region of *ONSEN* under HS because of the presence of cDNA made from *ONSEN*. Whether open chromatin is required for *ONSEN* insertion is also considered, and information on the insertion site is needed. Previous studies have shown that *ONSEN* in *nrpd1* mutants exhibits random insertions (Ito et al., 2016). Investigating the open chromatin of heat-stressed random insertion sites is a challenge.

siRNAs are closely associated with gene silencing (Hamilton et al., 2002). *Pol IV* transcribes TE- and repeat-related genes as primary transcripts that are loaded by RDR2 (RNA-dependent RNA polymerase 2), DCL3 (Dicer-like 3), and AGO4 (ARGONAUTE 4) proteins to produce siRNAs (Ferrafiat et al., 2019). Transgenerational transposition of *ONSEN* often occurs in *nrpd1* mutants (Ito et al., 2011; Ito et al., 2016). Northern blot analysis showed that, although *ONSEN* siRNAs were not observed in *suvh2* and *suvh2/9* under non-stress conditions, new siRNAs were synthesized after heat stress (**Figure 5B**). In contrast, deletion of SUVH9 did not disrupt siRNA synthesis under non-stress or heat-stress conditions. This indicated that the absence of SUVH2 affected the siRNA synthesis activity of *ONSEN*.

In conclusion, our results showed that SUVH2 is involved in the transcriptional silencing of *ONSEN* in a siRNA pathway-dependent manner. We provide evidence that SUVH2 and SUVH9 are functionally non-redundant when involved in regulating retrotransposon activity. In addition, plants can adapt their protective mechanisms to cope with the possibility of genetic disruption resulting from adversity.

## Data availability statement

The original contributions presented in the study are included in the article/**Supplementary Material**. Further inquiries can be directed to the corresponding author.

## Author contributions

XN: Writing – original draft. ZG: Writing – original draft. HI: Writing – original draft, Writing – review & editing.
