# Supplementary material for: Regulatory mechanism of heat-active retrotransposons by the SET domain protein SUVH2
Source: Front Plant Sci. 2024 Feb 8;15:1355626. doi: 10.3389/fpls.2024.1355626 (PMC10883384; doi:10.3389/fpls.2024.1355626)
Supplement: Supplementary file 1 [file Presentation_1.pptx]

## Slide 1
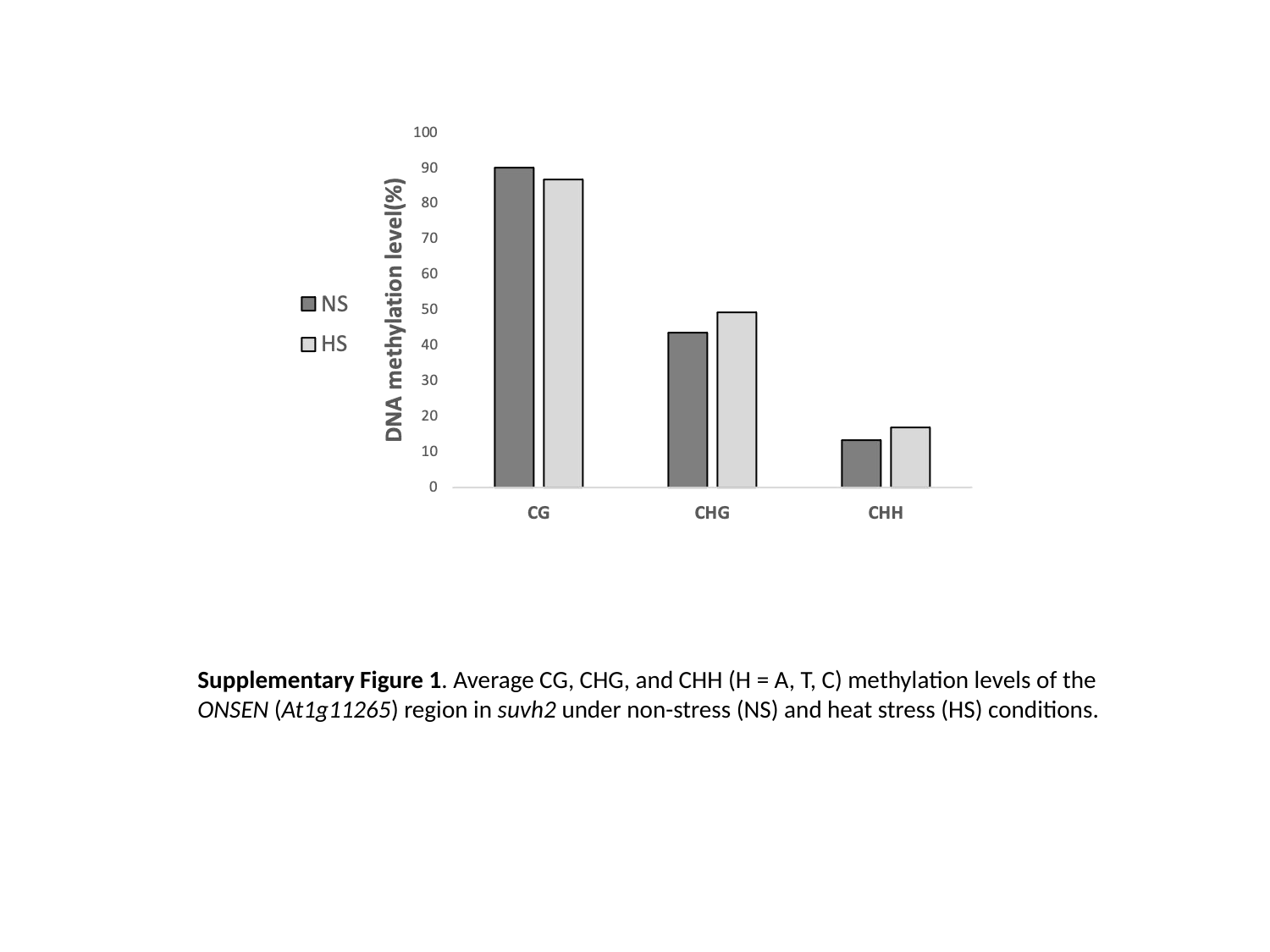

Supplementary Figure 1. Average CG, CHG, and CHH (H = A, T, C) methylation levels of the ONSEN (At1g11265) region in suvh2 under non-stress (NS) and heat stress (HS) conditions.

## Slide 2
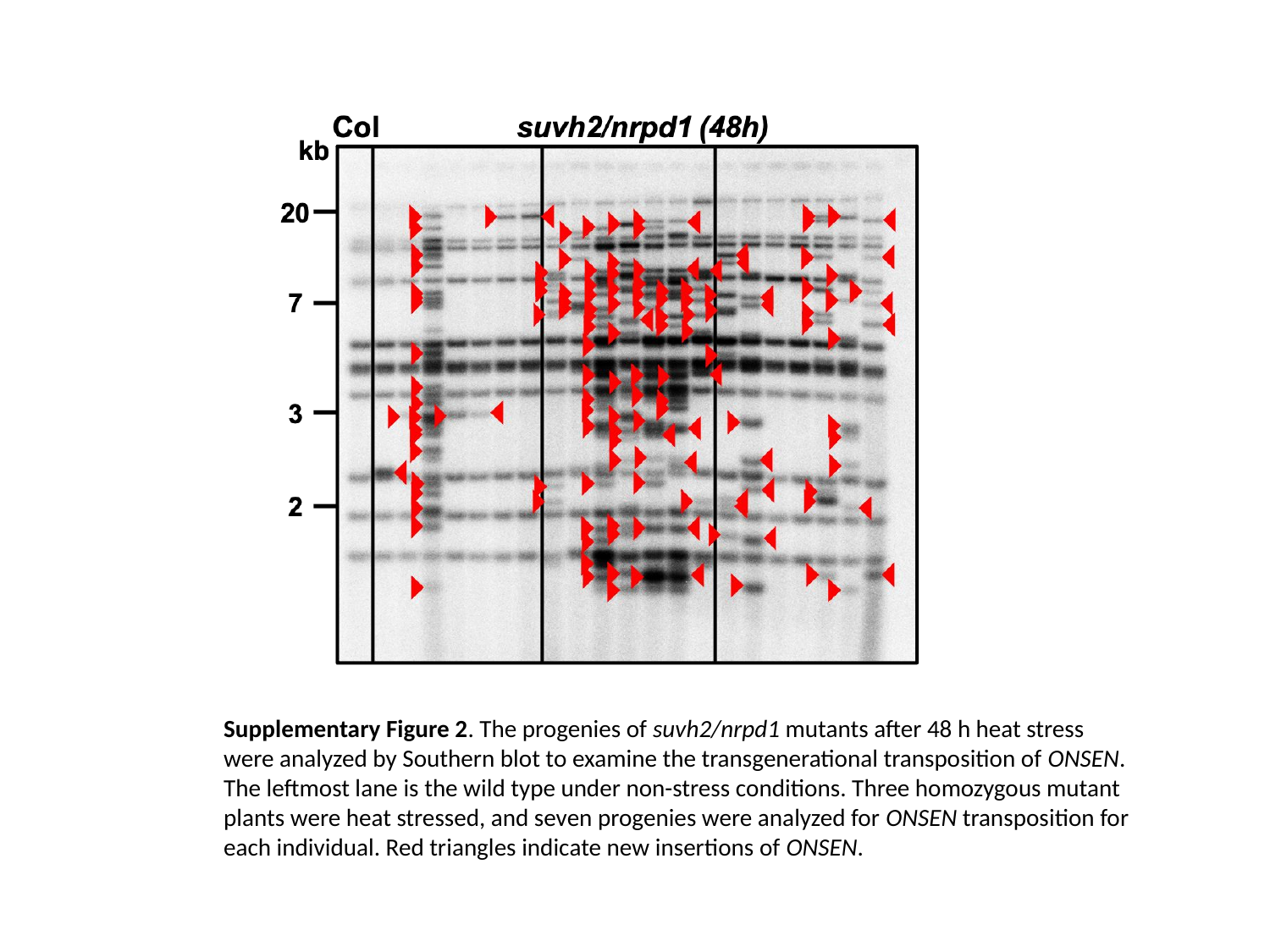

Supplementary Figure 2. The progenies of suvh2/nrpd1 mutants after 48 h heat stress were analyzed by Southern blot to examine the transgenerational transposition of ONSEN. The leftmost lane is the wild type under non-stress conditions. Three homozygous mutant plants were heat stressed, and seven progenies were analyzed for ONSEN transposition for each individual. Red triangles indicate new insertions of ONSEN.

## Slide 3
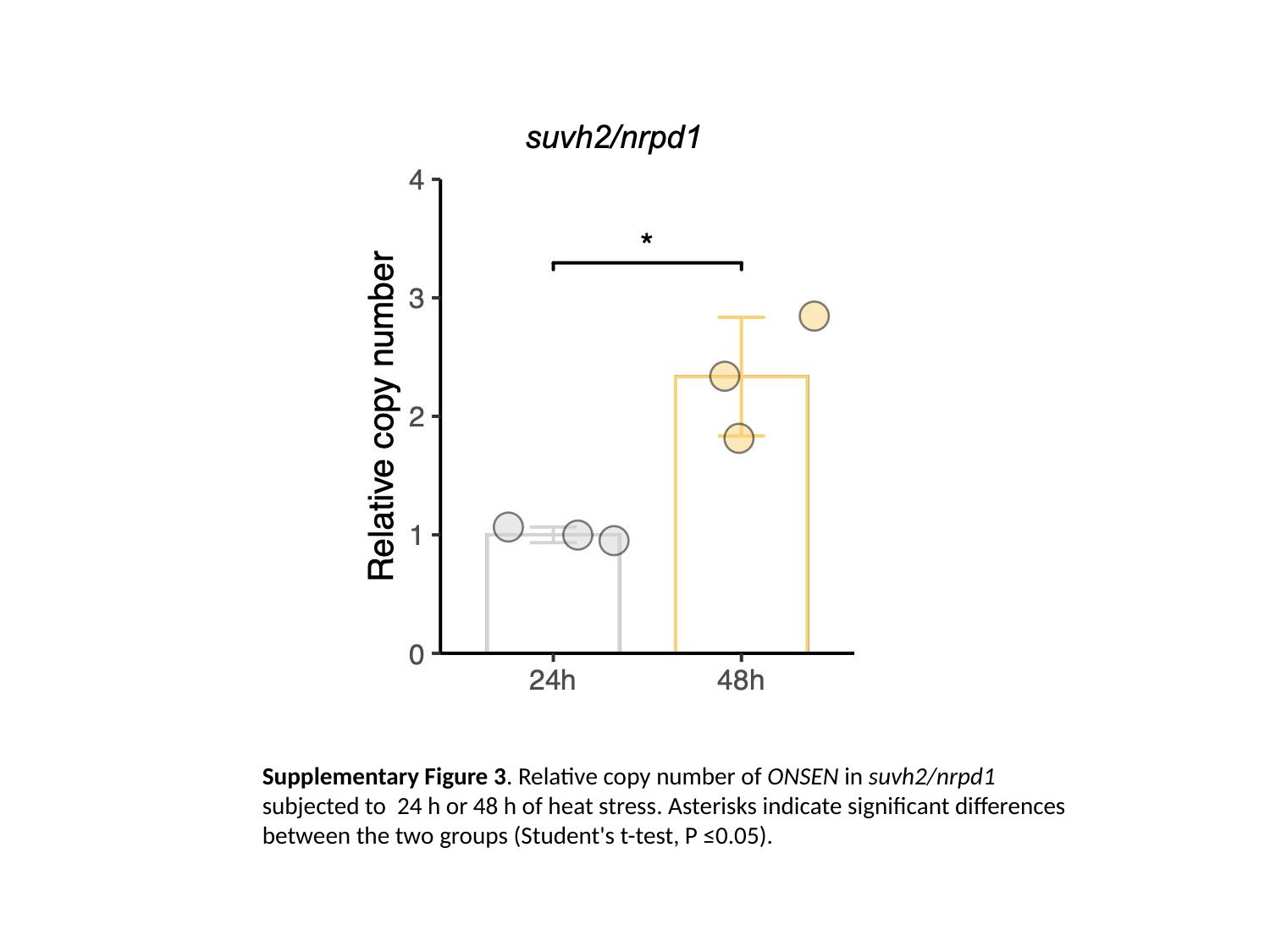

Supplementary Figure 3. Relative copy number of ONSEN in suvh2/nrpd1 subjected to 24 h or 48 h of heat stress. Asterisks indicate significant differences between the two groups (Student's t-test, P ≤0.05).

## Slide 4
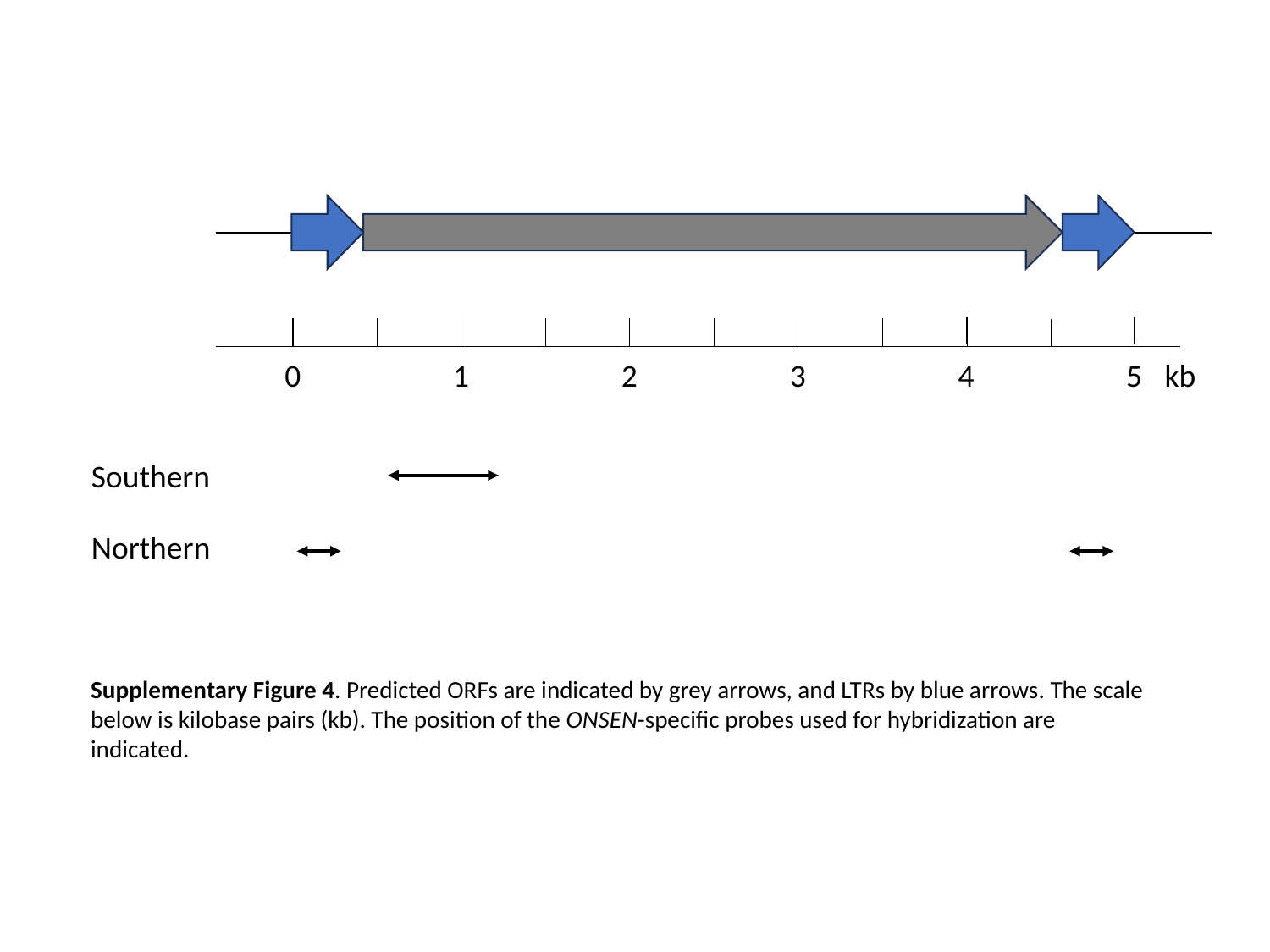

0
1
2
3
4
5
kb
Southern
Northern
Supplementary Figure 4. Predicted ORFs are indicated by grey arrows, and LTRs by blue arrows. The scale below is kilobase pairs (kb). The position of the ONSEN-specific probes used for hybridization are indicated.
