# Supplementary material for: Regulatory mechanism of heat-active retrotransposons by the SET domain protein SUVH2
Source: Front Plant Sci. 2024 Feb 8;15:1355626. doi: 10.3389/fpls.2024.1355626 (PMC10883384; doi:10.3389/fpls.2024.1355626)
Supplement: Supplementary file 2 [file Presentation_2.pptx]

## Slide 1
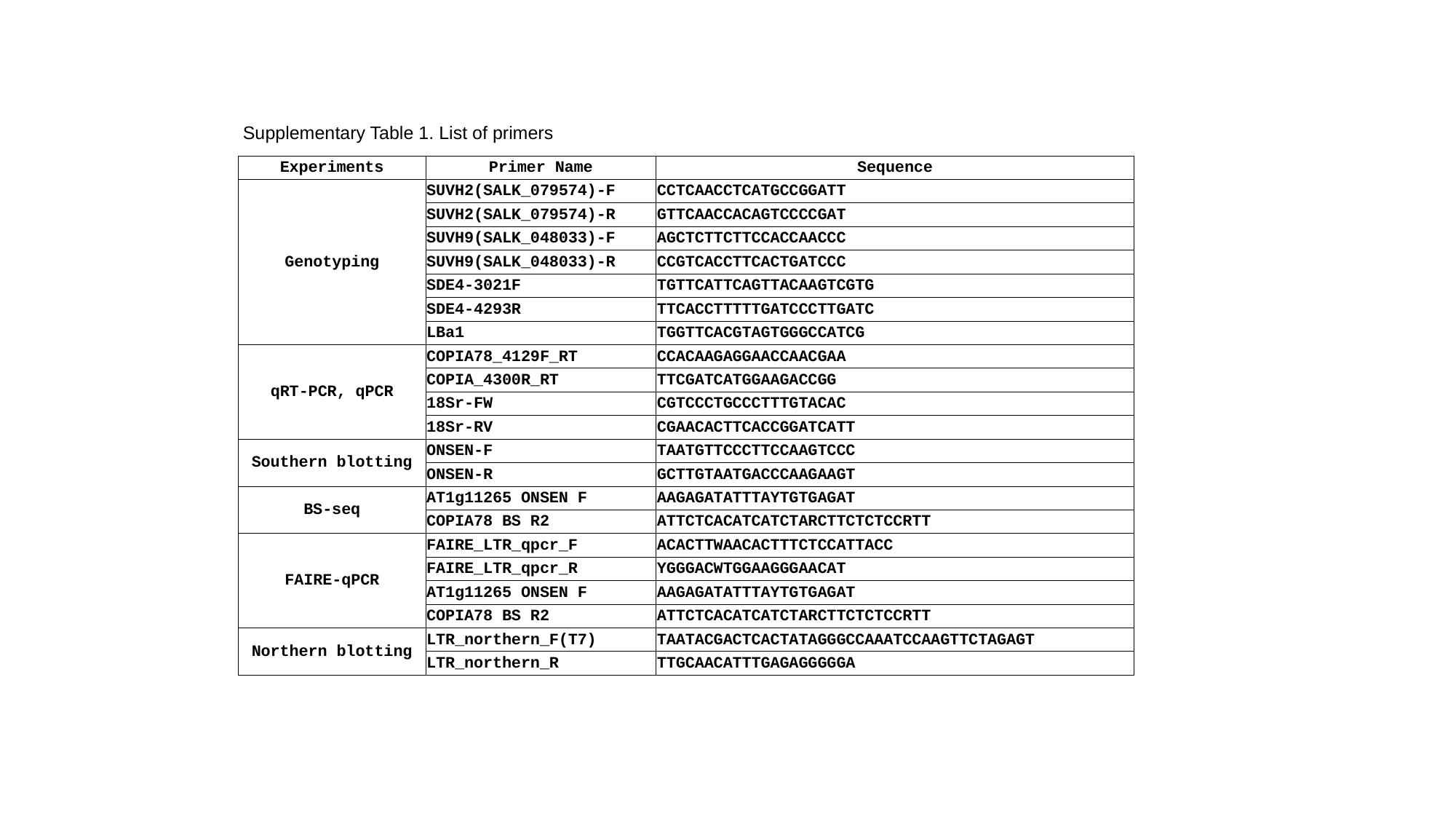

Supplementary Table 1. List of primers
| Experiments | Primer Name | Sequence |
| --- | --- | --- |
| Genotyping | SUVH2(SALK\_079574)-F | CCTCAACCTCATGCCGGATT |
| | SUVH2(SALK\_079574)-R | GTTCAACCACAGTCCCCGAT |
| | SUVH9(SALK\_048033)-F | AGCTCTTCTTCCACCAACCC |
| | SUVH9(SALK\_048033)-R | CCGTCACCTTCACTGATCCC |
| | SDE4-3021F | TGTTCATTCAGTTACAAGTCGTG |
| | SDE4-4293R | TTCACCTTTTTGATCCCTTGATC |
| | LBa1 | TGGTTCACGTAGTGGGCCATCG |
| qRT-PCR, qPCR | COPIA78\_4129F\_RT | CCACAAGAGGAACCAACGAA |
| | COPIA\_4300R\_RT | TTCGATCATGGAAGACCGG |
| | 18Sr-FW | CGTCCCTGCCCTTTGTACAC |
| | 18Sr-RV | CGAACACTTCACCGGATCATT |
| Southern blotting | ONSEN-F | TAATGTTCCCTTCCAAGTCCC |
| | ONSEN-R | GCTTGTAATGACCCAAGAAGT |
| BS-seq | AT1g11265 ONSEN F | AAGAGATATTTAYTGTGAGAT |
| | COPIA78 BS R2 | ATTCTCACATCATCTARCTTCTCTCCRTT |
| FAIRE-qPCR | FAIRE\_LTR\_qpcr\_F | ACACTTWAACACTTTCTCCATTACC |
| | FAIRE\_LTR\_qpcr\_R | YGGGACWTGGAAGGGAACAT |
| | AT1g11265 ONSEN F | AAGAGATATTTAYTGTGAGAT |
| | COPIA78 BS R2 | ATTCTCACATCATCTARCTTCTCTCCRTT |
| Northern blotting | LTR\_northern\_F(T7) | TAATACGACTCACTATAGGGCCAAATCCAAGTTCTAGAGT |
| | LTR\_northern\_R | TTGCAACATTTGAGAGGGGGA |
